# IL-17 and IL-23 Inhibitors Have the Fastest Time to Meaningful Clinical Response for Plaque Psoriasis: A Network Meta-Analysis

**DOI:** 10.3390/jcm13175139

**Published:** 2024-08-29

**Authors:** Pushkar Aggarwal, Alan B. Fleischer

**Affiliations:** Department of Dermatology, University of Cincinnati College of Medicine, Cincinnati, OH 45267, USA; aggarwpr@mail.uc.edu

**Keywords:** network meta-analysis, PASI-75, PASI-90

## Abstract

**Background/Objectives**: Several treatment options with differing mechanisms exist for plaque psoriasis. The objective of this analysis was to compare the time to onset of action among the available systemic therapies for plaque psoriasis. **Methods**: Randomized controlled trials that investigated two or more therapeutics for the management of moderate to severe plaque psoriasis were included. A weighted average time for 50% of patients to reach PASI75 and PAI90 with each of the therapeutics was calculated. A network meta-analysis was performed to determine which therapeutics were significantly faster in time to meaningful clinical response than others. **Results**: IL-17 inhibitors had the shortest time to achieve PASI75 and PASI90 followed by risankizumab in the weighted mean analysis. In the meta-analysis, the fastest time to PASI75 was seen with bimekizumab, brodalumab and ixekizumab. No significant (*p* < 0.05) difference was seen in the time to meaningful clinical response between these drugs; however, bimekizumab was significantly faster in time to PASI75 among the remaining therapeutics. In the meta-analysis for PASI90, the fastest time was seen with ixekizumab, bimekizumab, risankizumab, secukinumab and guselkumab with no significant differences in between these therapeutics. However, bimekizumab was significantly faster than the remaining therapeutics for PASI90. **Conclusions**: IL-17 and IL-23 inhibitors may be considered as requiring the shortest time for meaningful clinical response in plaque psoriasis. In addition to the time to onset, the safety profile of each drug needs to be considered when deciding on a therapeutic to initiate.

## 1. Introduction

Psoriasis represents one of the most prevalent immune-mediated inflammatory conditions. The most common form of psoriasis is plaque psoriasis but more than 15% of patients will also develop psoriatic arthritis, which can lead to permanent musculoskeletal changes, and 10% will develop ocular involvement [1]. The prompt recognition and treatment of psoriasis is imperative to reduce the progression of the condition and reduce the burden of disease from psoriasis.

In the last few decades, the number of systemic therapeutic options available to dermatologists for the management of plaque psoriasis has rapidly grown. Available biologic therapies include TNF-α inhibitors (Infliximab, adalimumab, certolizumab pegol and etanercept), IL-23 inhibitors (ustekinumab, guselkumab, risankizumab and tildrakizumab) and IL-17 inhibitors (secukinumab, ixekizumab, brodalumab and bimekizumab). As such, dermatologists have the luxury of selecting between many different therapies; however, this also comes with the burden of trying to select the therapeutic option that is the most efficacious and safe, is covered by insurance and is quick to provide relief.

Two common endpoints in many clinical trials for plaque psoriasis are the percentage/number of patients that achieve a 75% or greater improvement from baseline (PASI75) and a 90% or greater improvement from baseline (PASI90) as these endpoints have been shown to lead to improvement in a patient’s quality of life [2]. As such, reaching PASI75 or PASI90 represents a clinically meaningful response for the patient. We hypothesize that rapid improvement in plaque psoriasis through achieving PASI75 or PASI90 may be a significant factor when a provider and patient are having a shared decision-making regarding management options.

Previously, systematic reviews have been performed to compare the time to onset of action of drugs used in plaque psoriasis [3,4,5]. However, the lack of head-to-head randomized controlled trials for each available therapeutic option combination limits the ability to determine if the differences noted in the systematic reviews for the mean time to improvement for each therapeutic are significant. A network meta-analysis can factor in indirect comparisons and determine if the differences are significant as long as there are a certain number of direct comparisons to connect the network between each of the therapeutic options [6]. The objective of this analysis was to compare the time to onset of action among the various systemic therapies available for plaque psoriasis using a weighted mean average analysis and a network meta-analysis.

## 2. Material and Methods

The MEDLINE [7] database was queried for randomized clinical trials in which immunomodulators, biologics or oral small molecule inhibitors were used for the treatment of moderate to severe plaque psoriasis. Published data from randomized clinical trials of drugs that are approved by the Food and Drug Administration (FDA) for plaque psoriasis were included. Search terms included plaque psoriasis, randomized clinical trial and PASI.

Studies that investigated other forms of psoriasis (e.g., nail psoriasis and pustular psoriasis) in addition to plaque psoriasis were excluded. In addition, for the anti-psoriasis therapeutics that have been approved by the FDA, only FDA-approved doses were included. Studies were filtered to those that included a graphical representation over time (weeks) on the percentage of patients that reached PASI75 or PASI90. A minimum of ten patients in each treatment arm was required. Because a comparator for each study is needed to perform a network meta-analysis and because in placebo treatment groups 50% of patients never achieved PASI75 or PASI90, clinical trials in which placebo was the only comparator were excluded. As such, studies were filtered to only those that had at an active comparator being analyzed in addition to the drug of interest (i.e., at least two active therapeutic options). Since the end-point measure of this analysis was time needed for 50% of patients to reach PASI75 or PASI90, studies in which both the drug of interest and the active comparator did not have 50% of patients reach PASI75 or PASI90 by the end of the study timeframe were excluded. Each study was screened independently by the authors to confirm that they met the inclusion criteria. The analysis is a retrospective analysis utilizing publicly available published data. As such, IRB approval and informed consent are not required.

The primary outcome for this analysis was the time in weeks it took for 50% of the patients to reach PASI75 or PASI90 for each therapeutic. WebPlotDigitizer (v4.7) [8] was utilized to determine this time based on each studies’ graphical representation depicting weeks on the abscissa and percentage of patients achieving PASI75 or PASI90 on the ordinate. The graph depicting time to PASI75 and PASI90 for each therapeutic was uploaded into WebPlotDigitizer and the time at which the line crossed 50% of patients reaching PASI75 or PASI90 was determined. A weighted average for the time for 50% of patients to reach PASI75 and PASI90 based on the number of patients in the clinical trials was calculated for each therapeutic. In addition, the two Bayesian network meta-analyses through Markov chain Monte Carlo simulations were performed (one for PASI75 and one for PASI90) using MetaInsight (v5.2.1) [9,10]. A Bayesian meta-analysis treats both the data and model parameters as random variables, unlike the frequentist meta-analysis. The Bayesian meta-analysis appropriately takes into account the uncertainty around the heterogeneity variance and also estimates the relative treatment effects allowing for ranking of the treatments being analyzed in the network [11,12]. As such, the Bayesian meta-analysis is considered to be a more accurate meta-analysis [11]. Bimekizumab was used as the comparator in the forest plots since bimekizumab had the fastest observed time for 50% of patients to reach PASI75 and, of the drugs analyzed, is the latest to be approved by the FDA.

## 3. Results

**Figure 1:**
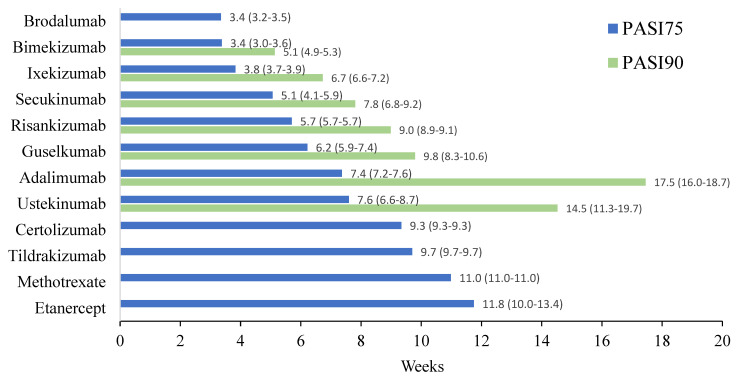
Comparison of time required for 50% of patients to reach PASI75 and PASI90 among therapeutics for moderate to severe plaque psoriasis. Data labels next to each horizontal bar contain the weighted average time required among all studies that met the inclusion criteria and the paratheses contain the range of time among these studies.

**Figure 2:**
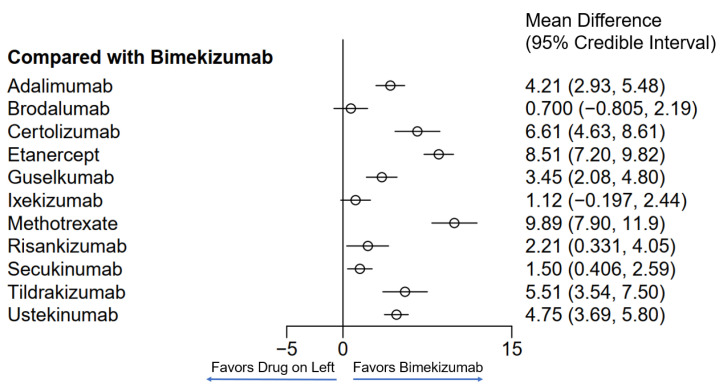
Network meta-analysis comparing psoriasis therapeutics with bimekizumab for PASI75. Bayesian 95% confidence interval is referred to as a credible interval.

**Figure 3:**
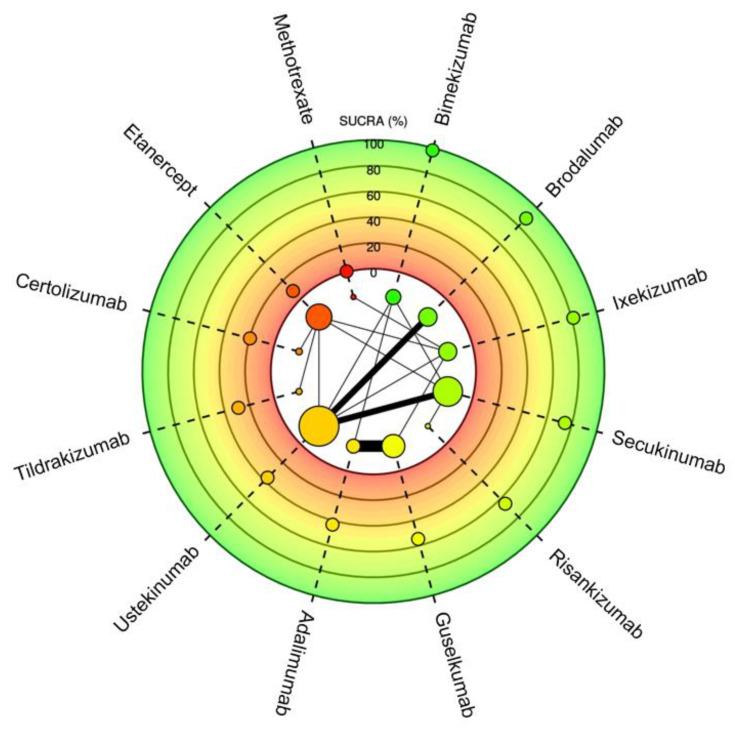
Meta-analysis network plot with a surface under the cumulative ranking curve (SUCRA) for PASI75 among therapeutics for plaque psoriasis. Higher SUCRA scores indicate faster time to reaching goal. Size of nodes represents number of participants and thickness of lines indicates number of trials conducted.

Twelve different drugs from nineteen studies met the inclusion criteria for PASI75 (Table 1). These drugs encompassed tumor necrosis factor alpha (TNF-α) inhibitors, interleukin (IL) 17 inhibitors, IL-23 inhibitors, IL-12/23 inhibitors and methotrexate. The weighted average analysis showed that the fastest time for 50% of patients to reach PASI75 was with the IL-17 inhibitors, specifically with brodalumab and bimekizumab tied at 3.4 weeks (Figure 1). Ixekizumab, secukinumab and risankizumab had the next fastest times, respectively. The slowest time for 50% of patients to reach PASI75 was seen with etanercept at 11.8 weeks followed by methotrexate at 11 weeks. The network meta-analysis revealed the fastest time with bimekizumab followed by brodalumab, ixekizumab and secukinumab, respectively (Figure 2 and Figure 3). No significant (*p* < 0.05) difference was seen between bimekizumab and brodalumab or between bimekizumab and ixekizumab. However, for all remaining therapies, bimekizumab was significantly quicker in reaching the endpoint. After the IL-17 inhibitors, risankizumab was noted to have the next fastest time. Methotrexate was the slowest and of the included biologics in the analysis, etanercept was the slowest in the meta-analysis.

**Figure 4:**
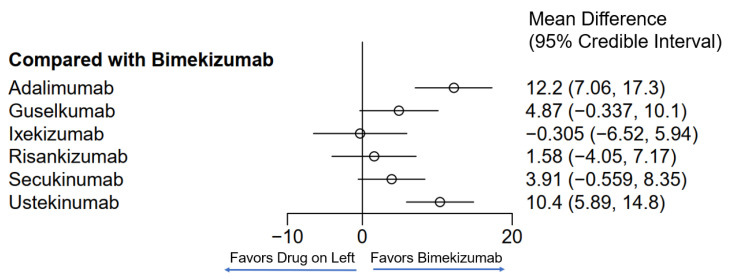
Network meta-analysis comparing psoriasis therapeutics with bimekizumab for PASI90. Bayesian 95% confidence interval is referred to as a credible interval.

**Figure 5:**
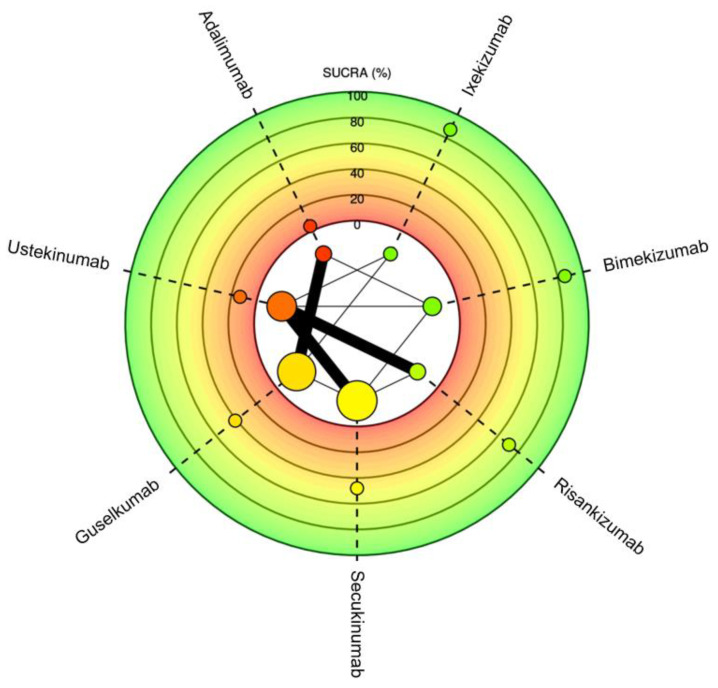
Meta-analysis network plot with a surface under the cumulative ranking curve (SUCRA) for PASI90 among therapeutics for plaque psoriasis. Higher SUCRA scores indicate faster time to reaching goal. Size of nodes represents number of participants and thickness of lines indicates number of trials conducted.

Seven different drugs from thirteen studies met the inclusion criteria for PASI90 (Table 1). There was representation from TNF-α inhibitors, IL-17 inhibitors, IL-23 inhibitors and IL-12/23 inhibitors; however, fewer drugs from each class met the requirements as compared to those that met for PASI75. The weighted average analysis showed bimekizumab as having the fastest time with 5.1 weeks followed by ixekizumab with 6.7 weeks and secukinumab with 7.8 weeks (Figure 1). The slowest times were with adalimumab at 17.4 weeks and ustekinumab at 14.5 weeks. The network meta-analysis revealed the fastest time with ixekizumab followed by bimekizumab and risankizumab (Figure 4 and Figure 5). However, no significant difference was noted between ixekizumab, bimekizumab, risankizumab, secukinumab and guselkumab. Compared to bimekizumab, adalimumab and ustekinumab were significantly slower in time for 50% of patients to reach PASI90.

## 4. Discussion

In 2019, Yao and Lebwohl [5] analyzed the time for 25% of patients to achieve PASI75 among adalimumab, infliximab, ustekinumab, etanercept, brodalumab, ixekizumab and secukinumab. Brodalumab was found to have the shortest time to achieve this outcome followed by ixekizumab, secukinumab, infliximab and adalimumab. In addition, in 2020, Egeberg and colleagues [4] found that brodalumab had the shortest time for 50% of patients to reach PASI90 followed by ixekizumab, secukinumab and risankizumab among IL-17 and IL-23 inhibitors. The current analysis builds upon the above studies by including clinical trials since 2020, including newly approved therapeutic options, such as bimekizumab, and conducting a network meta-analysis to determine which differences are significant. The results of this analysis are in agreement with Yao and Lebwohl’s and Egeberg’s analysis in that IL-17 inhibitors had the shortest time to achieve the outcome followed by risankizumab in the weighted mean analysis. Of the IL-17 inhibitors, bimekizumab and brodalumab were tied for the fastest time in the PASI75 analysis. However, for PASI75, the differences between bimekizumab and brodalumab and between bimekizumab and ixekizumab were not significant. For PASI90, the network meta-analysis showed the fastest time to onset with ixekizumab; however, the differences between ixekizumab, bimekizumab, risankizumab, secukinumab and guselkumab were not significant. Overall, the results from the network meta-analysis show that some of the differences seen in the weighted average analysis are not significant with the amount of current data available. More clinical trials with larger patient populations would help shrink the confidence intervals and allow us to further delineate which differences in time to clinical improvement among the above therapies are significant.

The pathway causing inflammation from psoriasis begins with genetic predisposition and/or a triggering environment factor that damages keratinocyte leading to release of interleukin 1, interleukin 6 and tumor necrosis factor α and also leading to the activation of dendritic cells [33,34]. These dendritic cells release cytokines, including interleukin 6 and transforming growth factor β, which causes T cells to differentiate towards Th17 cells. Dendritic cells also maintain this differentiation towards a Th17 phenotype with the release of interleukin 23. The Th17 cells release interleukin 17, which causes keratinocyte proliferation and the production of neutrophil-recruiting mediators and antimicrobial peptides [33,34]. IL-17 inhibitors may have a faster time to meaningful plaque psoriasis improvement due to the target IL-17 cytokine being more downstream in the pathway than IL-23 or TNF-α (targets of IL-23 and TNF-α inhibitors).

While network meta-analysis provides novel information through indirect comparisons, direct comparisons through head-to-head trials would provide more accurate data and less confounders due to variation in the population between clinical trials such as age, gender, comorbidities and ethnicity that can affect the time to psoriasis clearance [35]. Clinical trials involving some therapeutics, such as deucravacitinib and infliximab, did not meet the eligibility criteria in this analysis. As such, we were unable to compare these therapeutics. In addition to the time to onset, the safety profile of each drug and its contraindications need to be heavily weighed when deciding on a therapeutic to initiate for a patient.

## 5. Conclusions

When considering time to action of the various therapeutics available for plaque psoriasis, clinicians can consider bimekizumab, brodalumab and ixekizumab as having the fastest times to achieve PASI75 with no significant difference in between these drugs. However, bimekizumab was seen to be significantly faster in time to clinically meaningful response among the remaining therapeutics, including secukinumab, risankizumab, adalimumab and ustekinumab, among others. In the PASI90 analysis, ixekizumab, bimekizumab, risankizumab, secukinumab and guselkumab had the fastest times to achieving PASI90 and no significant differences were noted between these therapeutics. However, bimekizumab and ixekizumab were seen to be significantly faster than adalimumab and ustekinumab for PASI90. Taking into account both analyses, IL-17 and IL-23 inhibitors may be considered as requiring the shortest time for meaningful clinical improvement in plaque psoriasis.

## Figures and Tables

**Table 1:** Studies meeting the inclusion criteria for the PASI75 and PASI90 analysis.

Authors	Article Title	Met Criteria for PASI75 Analysis	Met Criteria for PASI90 Analysis
Bagel et al. [13]	Secukinumab maintains superiority over ustekinumab in clearing skin and improving quality of life in patients with moderate to severe plaque psoriasis: 52-week results from a double-blind phase 3b trial (CLARITY)	Yes	Yes
Blauvelt et al. [14]	Secukinumab is superior to ustekinumab in clearing skin of subjects with moderate-to-severe plaque psoriasis up to 1 year: Results from the CLEAR study	Yes	Yes
Blauvelt et al. [15]	Efficacy and safety of guselkumab, an anti-interleukin-23 monoclonal antibody, compared with adalimumab for the continuous treatment of patients with moderate to severe psoriasis: Results from the phase III, double-blinded, placebo- and active comparator-controlled VOYAGE 1 trial	Yes	Yes
Blauvelt et al. [16]	A head-to-head comparison of ixekizumab vs. guselkumab in patients with moderate-to-severe plaque psoriasis: 24-week efficacy and safety results from a randomized, double-blinded trial	Yes	Yes
de Vries et al. [17]	A prospective randomized controlled trial comparing infliximab and etanercept in patients with moderate-to-severe chronic plaque-type psoriasis: the Psoriasis Infliximab vs. Etanercept Comparison Evaluation (PIECE) study	Yes	No
Gordon et al. [18]	A Phase 2 Trial of Guselkumab versus Adalimumab for Plaque Psoriasis	Yes	No
Gordon et al. [19]	Efficacy and safety of risankizumab in moderate-to-severe plaque psoriasis (**UltIMMa-1** and UltIMMa-2): results from two double-blind, randomised, placebo-controlled and ustekinumab-controlled phase 3 trials	No	Yes
Gordon et al. [19]	Efficacy and safety of risankizumab in moderate-to-severe plaque psoriasis (UltIMMa-1 and **UltIMMa-2**): results from two double-blind, randomised, placebo-controlled and ustekinumab-controlled phase 3 trials	No	Yes
Griffiths et al. [20]	Comparison of ixekizumab with etanercept or placebo in moderate-to-severe psoriasis (UNCOVER-2 and UNCOVER-3): results from two phase 3 randomised trials	Yes	No
Langley et al. [21]	Secukinumab in plaque psoriasis--results of two phase 3 trials	Yes	No
Lebwohl et al. [22]	Phase 3 Studies Comparing Brodalumab with Ustekinumab in Psoriasis (AMAGINE-2)	Yes	No
Lebwohl et al. [22]	Phase 3 Studies Comparing Brodalumab with Ustekinumab in Psoriasis (AMAGINE-3)	Yes	No
Lebwohl et al. [23]	Certolizumab pegol for the treatment of chronic plaque psoriasis: Results through 48 weeks of a phase 3, multicenter, randomized, double-blind, etanercept- and placebo-controlled study (CIMPACT)	Yes	No
Reich et al. [24]	Efficacy and safety of guselkumab, an anti-interleukin-23 monoclonal antibody, compared with adalimumab for the treatment of patients with moderate to severe psoriasis with randomized withdrawal and retreatment: Results from the phase III, double-blind, placebo- and active comparator-controlled VOYAGE 2 trial	Yes	Yes
Reich et al. [25]	Comparison of ixekizumab with ustekinumab in moderate-to-severe psoriasis: 24-week results from IXORA-S, a phase III study	Yes	Yes
Reich et al. [26]	Tildrakizumab versus placebo or etanercept for chronic plaque psoriasis (reSURFACE 1 and reSURFACE 2): results from two randomised controlled, phase 3 trials	Yes	No
Reich et al. [27]	A 24-week multicentre, randomized, open-label, parallel-group study comparing the efficacy and safety of ixekizumab vs. fumaric acid esters and methotrexate in patients with moderate-to-severe plaque psoriasis naive to systemic treatment	Yes	No
Reich et al. [28]	Bimekizumab versus ustekinumab for the treatment of moderate to severe plaque psoriasis (BE VIVID): efficacy and safety from a 52-week, multicentre, double-blind, active comparator and placebo controlled phase 3 trial	Yes	Yes
Reich et al. [29]	Bimekizumab versus Secukinumab in Plaque Psoriasis	Yes	Yes
Reich et al. [30]	Guselkumab versus secukinumab for the treatment of moderate-to-severe psoriasis (ECLIPSE): results from a phase 3, randomised controlled trial	No	Yes
Warren et al. [31]	Efficacy and safety of risankizumab vs. secukinumab in patients with moderate-to-severe plaque psoriasis (IMMerge): results from a phase III, randomized, open-label, efficacy-assessor-blinded clinical trial	Yes	Yes
Warren et al. [32]	Bimekizumab versus Adalimumab in Plaque Psoriasis	Yes	Yes

## Data Availability

Data can be provided upon request to the authors.
